# SET ESG ratings and firm value: The new sustainability performance assessment tool in Thailand

**DOI:** 10.1371/journal.pone.0315935

**Published:** 2025-02-07

**Authors:** Mongkhol Moolkham

**Affiliations:** School of Interdisciplinary Studies, Mahidol University, Kanchanaburi, Thailand; Lucian Blaga University of Sibiu: Universitatea Lucian Blaga din Sibiu, ROMANIA

## Abstract

This study investigates the influence of SET ESG ratings on firm value of companies listed on the Stock Exchange of Thailand (SET), emphasizing the multifaceted relationships between ESG performance, third-party evaluations, and market reactions following SET ESG ratings announcements. The findings reveal that firms with higher SET ESG ratings experience significant enhancements in firm value due to their superior management of environmental and social risks, which reduces regulatory and reputational threats. Furthermore, the research uncovers the nuanced role of third-party ESG evaluations, indicating that while endorsements from reputable sources can amplify the positive effects of SET ESG ratings, more or conflicting assessments can lead to diminished firm value. Specifically, a single strong endorsement markedly enhances firm value, whereas evaluations from two to three third parties may introduce confusion, leading to increased information asymmetry. This finding challenges the assumption that multiple endorsements always translate to greater value, emphasizing the need for firms to strategically select their evaluators to optimize market perceptions. Moreover, the study illustrates that announcements of SET ESG ratings result in immediate and sustained increases in firm value, highlighting ESG performance as a critical determinant of long-term viability in the eyes of investors. These results provide actionable guidance for investors and policymakers, advocating for the integration of robust ESG practices to enhance market confidence and drive long-term value creation. This research contributes to the existing literature by elucidating the intricate dynamics between SET ESG ratings, third-party evaluations, and firm value, thereby offering valuable perspectives for firms navigating a sustainability-focused landscape.

## Introduction

The world is facing various pressing issues, such as climate change, resource depletion, and societal contradictions, underscoring the urgency and challenge of achieving sustainable development on a global level [1]. Sustainable development involves fostering economic growth, social inclusion, and environmental protection in a balanced and integrated manner, ensuring that development efforts are environmentally friendly, socially equitable, and economically viable over the long term. Consequently, sustainability is increasingly acknowledged as a pivotal aspect of overall value, complementing financial and economic metrics within firms [2]. As a result, firms face mounting pressure, both internally and externally, to enhance their non-financial performance, necessitating strategies to mitigate adverse environmental and social impacts to align with evolving societal expectations [3]. Moreover, the transition towards a more sustainable financial market necessitates investor transparency and trust, objectives pursued through sustainable finance disclosure regulations [4].

During the 1990s, the Financial Initiative of the United Nations Environment Program (UNEP) introduced the concept of Environmental, Social, and Governance (ESG) factors as integral components in making investment decisions. Since then, investors have increasingly focused on environmental preservation and corporate governance, resulting in a growing influence of ESG performance on investment choices [5]. ESG activities encompass environmental and social practices that extend beyond a company’s primary economic objectives, attracting considerable attention from regulators, investors, and stakeholders [6]. Resource-based and stakeholder theories postulate that ESG practices represent ethical imperatives that confer competitive advantages to organizations while strengthening their relationships with stakeholders [7]. Furthermore, ESG is widely recognized globally as crucial for achieving sustainable development goals [8]. This heightened attention to ESG performance has prompted a notable shift in investment behavior, with an increasing number of investors considering non-financial parameters alongside traditional financial indicators [9]. The integration of ESG considerations into corporate investment decisions signifies a commitment to responsible, long-term investment practices—a topic that has garnered considerable scholarly attention [10]. Additionally, ESG has emerged as a modern method for assessing corporate risk among contemporary investors [11]. Unlike conventional financial performance evaluations, ESG assessments focus on a company’s environmental, social, and governance factors, serving as benchmarks for evaluating firm development potential and investor compliance [12]. At the corporate level, the importance of ESG lies in the ability of managers to enhance ESG performance not only to attract conscientious investors but also as a strategy to bolster investor confidence and improve the company’s reputation for financial transparency and reliability [13]. Moreover, robust ESG policies can be regarded as significant supplementary factors and justifications for stock market results. Effective stakeholder management within organizations can enhance trust, leading to improved financial performance and overall stock market outcomes [14]. The establishment of supportive policies and legislation that incentivize corporations to adopt and report effective ESG practices can enhance stock market stability and liquidity while fostering long-term economic growth [15]. Furthermore, ESG performance has bolstered investor confidence, consequently enhancing firm value [16]. Although ESG investments may be burdensome and more costly for businesses, they can attract recognition in capital markets and lead to price premiums [17].

In the process of investment decision-making, ESG ratings are increasingly recognized as pivotal reference metrics [1]. Typically, ESG ratings are either segmented into separate scores for each of the three components or consolidated into a unified rating [18]. These ratings serve the purpose of mitigating information asymmetry and enhancing corporate governance, particularly in markets characterized by a significant number of individual investors and relatively limited investor protection [19]. The disclosure of the financial implications of ESG ratings provides invaluable insights for socially conscious investors striving to maximize portfolio returns [20]. Additionally, ESG ratings play a critical role in attracting investors and influencing market dynamics, aiding firms in cultivating a favorable societal image and yielding positive returns. Participation in ESG activities not only produces favorable financial outcomes but also contributes to the stabilization of capital markets by attracting investments associated with sustainable practices. Firms that adopt ESG practices are perceived as bearing lower risk levels and may experience diminished volatility [21]. However, past research also provides insights into the negative impact of ESG on stock performance, indicating that firms with lower ESG ratings may yield higher returns compared to their higher-rated counterparts [22]. Moreover, ESG scores have been found to exert an insignificant influence on investor returns, with stocks of lower ESG-rated companies offering greater protection than those with higher ESG scores. This phenomenon can be attributed to increased investor interest in ESG stocks, which may lead to overpricing and potential underpricing of lower-rated stocks [23]. Furthermore, an increase in the ESG index has been shown to decrease investor sentiment, while a decline in ESG index performance leads to an uptick in investor sentiment, suggesting investor indifference toward firms’ ESG actions [24]. Additionally, past research indicates that regardless of market conditions, firms with high ESG ratings do not consistently outperform their lower-rated counterparts [25]. While enhancing ESG practices can improve public support for a firm’s policies, stakeholders may perceive ESG operations as redundant and prefer to invest in innovation, potentially resulting in decreased firm value [26]. Furthermore, it is emphasized that investor engagement in ESG factors should not take precedence over engagement in other value drivers, as investors seek exceptional companies, not merely those excelling in ESG [27]. The lack of consensus on ESG concepts has led to ambiguity regarding their impact on investors and markets, hindering the interpretation of empirical data and the formulation of effective regulations [28]. Moreover, while firms with higher ESG ratings often enjoy a competitive edge, there is scant research on the spillover effects of these ratings [29]. Additionally, the influence of ESG performance on market valuation via investor sentiment in emerging markets remains unclear [16]. Consequently, further investigation is warranted to assess the implications of ESG, particularly within emerging economies and various industries worldwide, to facilitate valuable and constructive comparisons [30].

This study seeks to examine the impact of sustainability performance on firm value through the use of SET ESG ratings, a new evaluation tool developed by the Stock Exchange of Thailand, specifically designed to assess the sustainability performance of Thai firms. By analyzing how investors perceive and incorporate these ratings into their investment decisions within the Thai stock market, this research significantly contributes to the understanding of sustainability performance in the context of an emerging market. Unlike existing literature, which predominantly focuses on developed markets, this study explicitly addresses a critical gap by emphasizing the unique contributions of the SET’s sustainability performance assessment tool, which is tailored to local conditions. By integrating local regulatory, economic, and social factors, this tool effectively confronts the distinct challenges faced by Thai firms, thereby enhancing the relevance of sustainability metrics in evaluating firm value in emerging markets. The findings illustrate how SET ESG ratings can positively influence firm value and underscore the importance of regionally tailored sustainability performance in fostering sustainable investment strategies. This alignment enables firms to pursue global sustainability objectives while adapting to Thailand’s specific regulatory landscape. By addressing this significant research gap, this study offers valuable insights for policymakers, investors, and corporate leaders, enriching the understanding of how sustainability practices impact firm value in areas that have been less extensively studied, thereby advancing the discourse on sustainable finance in emerging markets.

## Literature review

According to agency theory, conflicts of interest often arise between management and shareholders due to differing objectives and the information asymmetry that characterizes their relationship [31]. This theory lays the groundwork for understanding how a misalignment of interests can negatively impact firm value and stakeholder trust. In response, signaling theory offers a complementary perspective by suggesting that organizations can mitigate this information asymmetry through credible signaling of their attributes and intentions [32]. In the context of ESG practices, firms can leverage their sustainability initiatives as credible signals to assure stakeholders of their commitment to responsible management and ethical governance. Transitioning to stakeholder approach theory, which broadens the focus beyond shareholders to include all parties affected by a firm’s operations [33], establishes a critical link to ESG practices. This perspective emphasizes that firms must consider the interests of various stakeholders—including employees, customers, and the community—to achieve long-term success. By aligning ESG efforts with stakeholder expectations, organizations not only address agency conflicts but also foster a more inclusive business model that enhances overall firm value. Furthermore, this discussion aligns with legitimacy theory, which posits that firms strive to gain public recognition and support by conforming to societal norms and values [31]. In this light, ESG practices can be viewed as essential for firms seeking legitimacy in the eyes of their stakeholders. By aligning operations with societal expectations, firms can improve their reputational capital and gain competitive advantages. Additionally, institutional theory contributes to this framework by asserting that social institutions—such as laws, norms, and cultural values—exert significant influence over organizational behavior. ESG disclosure can therefore be seen as strategic responses to these institutional pressures [34], demonstrating a firm’s commitment to sustainability and responsible governance. This interaction between institutional factors and corporate behavior further highlights the importance of ESG practices in navigating the complexities of modern business environments. This study extends and challenges existing theories by illustrating how organizations can utilize sustainability performance as a tool to bridge the information gap between management and stakeholders. It emphasizes the critical role of non-financial performance metrics, which are frequently undervalued in traditional investment evaluations. By prioritizing ESG considerations, firms not only align with institutional norms and gain legitimacy but also enhance their reputations and operational efficiencies, attract socially responsible investors, and elevate overall firm valuation. In conclusion, this research posits that the integration of these theoretical frameworks—agency theory, signaling theory, stakeholder theory, legitimacy theory, and institutional theory—provides a comprehensive understanding of the multifaceted benefits of ESG practices. By doing so, it highlights the necessity for firms to actively engage in sustainable practices as a strategic imperative, ultimately advancing the discourse on the value of non-financial metrics in driving firm success in today’s interconnected and socially conscious market landscape.

**Fig 1:**
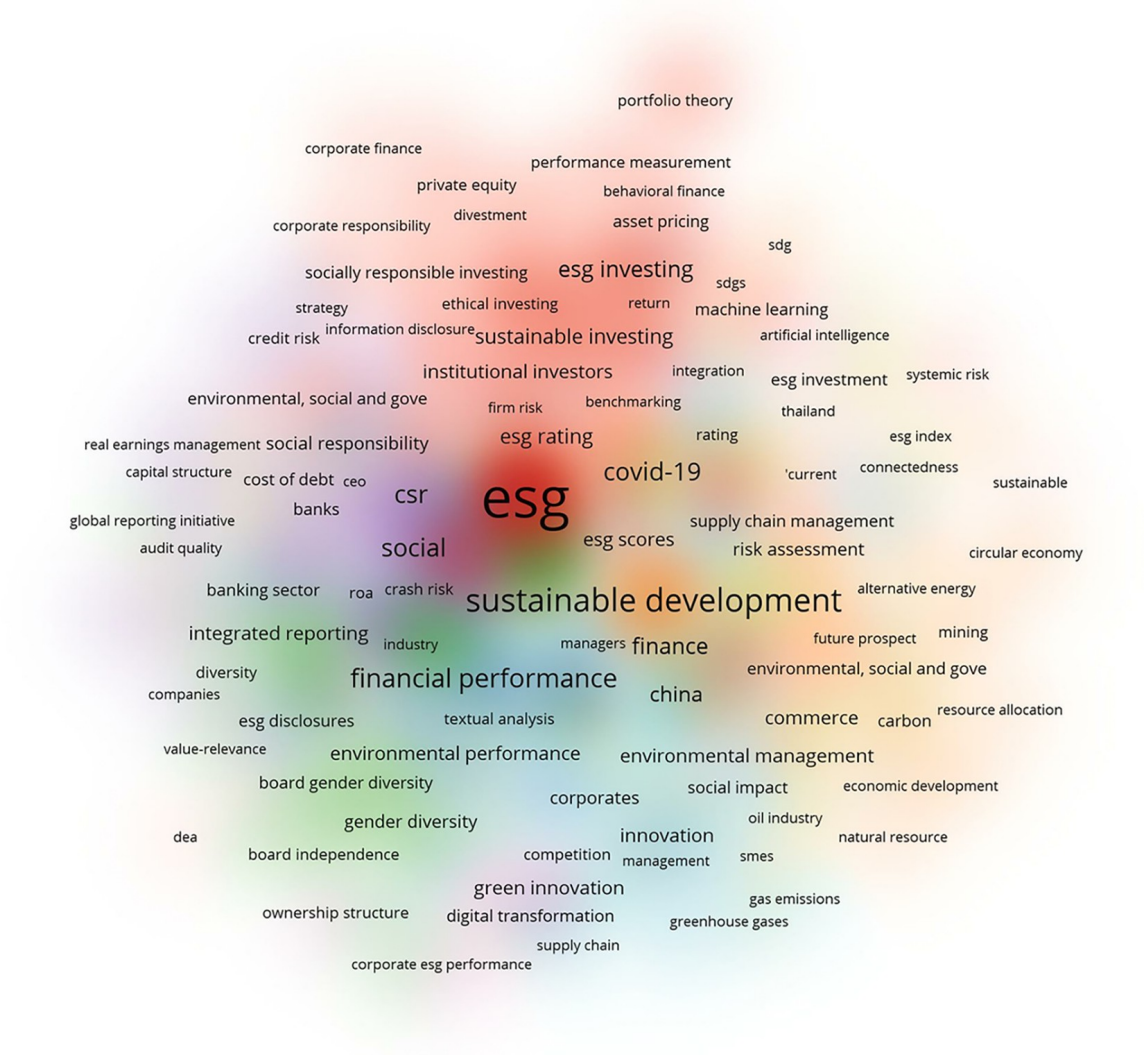
Bibliographic coupling.

As the significance and relevance of ESG practices for governments, investors, and stakeholders grew, the academic community was encouraged to conduct extensive, renewed, and more in-depth research, which is evidenced by the increasing quantity of publications devoted to ESG reports and the variety of methodologies used to analyze them [35], as shown in Fig 1. Firm ESG performance, as a systematic process, focuses on maximizing social welfare while concurrently pursuing economic benefits for corporations. Moreover, a favorable ESG profile is positively associated with the stock price response to new information conveyed through changes in credit ratings. Signaling theory [36] indicates that firms can leverage ESG ratings as credible signals to positively distinguish themselves to investors. Consequently, when a stock’s ESG characteristics are assessed as high or improving, investors may reward them in the event of a credit rating upgrade, penalize them less in the event of a downgrade, or both [37]. Past studies have also advocated incorporating ESG considerations into investment strategies to reduce potential ESG risk exposure. It emphasizes increasing investors’ understanding of ESG risks and opportunities [38]. Furthermore, stronger investor sentiment towards high ESG scores tends to raise their asset holdings and prices, while increased perceived ESG ambiguity has a negative impact on these indicators [39].

The onset of the COVID-19 pandemic has stimulated heightened interest in studying economic sustainability and ESG factors across both academic and industrial sectors [40]. Numerous studies have delved into the role of ESG in augmenting business performance amid the pandemic. Past research findings also indicate that robust ESG performance contributed to bolstering stock market stability and enhancing market liquidity in Japan during the COVID-19 crisis [15]. Moreover, strong ESG performance has been found to mitigate stock price fluctuations stemming from the pandemic’s impact, thereby enhancing resilience and preserving stability in stock prices in both China [41] and the U.S. [42]. Additionally, it was revealed that ESG performance on equities in emerging markets, such as India, played a role in dampening the volatility of stock returns amidst the COVID-19 pandemic [43], and the application of ESG principles can also improve the effectiveness of company investments by mitigating information asymmetries and conflicts of interest [44].

Environmental concerns and economic considerations shape investors’ attitudes and intentions towards equity investment, supported by ESG criteria [45]. High ESG scores wield substantial influence on investor reactions to positive information, amplifying stock price reactions [37]. Moreover, ESG ratings exert an impact on the excess returns of stocks [1]. Research conducted in China revealed that ESG performance positively affects stock returns [46]. A higher ESG rating reduces idiosyncratic stock risk, with this effect being more pronounced for stocks with higher ESG ratings. However, even equities with lower ESG ratings exhibit significantly lower idiosyncratic risk compared to those without an ESG rating. These findings support the notion that obtaining an ESG rating diminishes uncertainty regarding the future risk and return of stocks [47]. Additionally, ESG ratings exert a significant influence on the stock prices of insurance companies, with upgrades resulting in price increases and downgrades leading to declines [48]. ESG performance also has a negative correlation with stock price fragility, implying that enhanced ESG performance decreases stock price fragility [49]. Elevated ESG ratings reduce the probability of stock price crashes [50]. Furthermore, ESG performance contributes to bolstering the liquidity of a firm’s stock by mitigating corporate risk and eliciting support from stakeholders [51]. Firms with higher ESG ratings have a more pronounced response to earnings news. In addition, robust ESG practices can help reduce the negative effects of poor earnings, enhancing the resilience of companies with high ESG ratings against negative market reactions [52]. Finally, the past study also proposes that governments should establish ESG ratings to incentivize firms to focus on disclosing their ESG performance. Such ratings can aid investors in assessing a company’s sustainability prospects, and fostering ESG operations can enhance the economic sustainability of firms, particularly in developing countries [53].

For these reasons, this study therefore hypothesizes that:

*H1*: *SET ESG ratings have a positive impact on firm value of companies listed on the Stock Exchange of Thailand*.

This hypothesis posits that investors form perceptions regarding a firm’s capacity to effectively manage environmental and social risks, particularly in relation to news about ESG ratings. It is anticipated that positive announcements concerning a firm’s ESG ratings will enhance investor confidence, leading to an increase in firm value as investors recognize the firm’s commitment to sustainability and responsible governance. Conversely, negative news related to ESG ratings may diminish investor confidence, resulting in decreased stock prices as the perceived risk associated with environmental and social challenges rises. Ultimately, the study expects to demonstrate a significant impact of ESG ratings on investor behavior, illustrating how perceptions of ESG performance influence market dynamics in the context of sustainability performance in emerging markets.

## Methodology

This study examines the impact of SET ESG ratings on firm value of companies listed on the Stock Exchange of Thailand that were SET ESG-rated in 2023, encompassing a total of 175 firms (excluding companies listed on the Market for Alternative Investment: MAI). To mitigate potential confounding factors affecting the results, the analysis is limited to firms with fiscal years concluding on December 31 and excludes those experiencing trading suspensions, lacking complete data, or having been removed from the SET ESG ratings list. Consequently, the final sample consists of 157 firms. Given that the SET ESG ratings were announced for the first time in 2023, this research focuses exclusively on short-term outcomes. The evaluation of firm value following the ESG ratings announcement is based on daily stock price data collected from November 6, 2023 (the date of the SET ESG rating announcement) to December 28, 2023 (the final trading day of the year), yielding 5,809 firm-year observations. Data pertaining to the SET ESG ratings and relevant financial metrics are obtained from the database of the Stock Exchange of Thailand, a key regulatory authority in Thailand (refer to SET ESG ratings at https://setsustainability.com, and financial information of companies listed at https://www.setsmart.com). This comprehensive methodology ensures that the study accurately captures the immediate effects of SET ESG ratings on firm value while accounting for the specific context of the Thai market.

Table 1 outlines the definitions of the variables employed in this study, which are critical for investigating the relationship between SET ESG ratings and firm value. The dependent variable, firm value, is measured through market capitalization, a widely accepted proxy in similar research due to its broad reflection of market sentiment and company performance. The independent variable, SET ESG rating, evaluates a firm’s adherence to environmental, social, and governance principles, which is hypothesized to influence investor sentiment and, subsequently, firm value. The selection of these variables is informed by prior studies that consistently demonstrate the significant impact of ESG performance on firm value, particularly in emerging markets, such as Korean firms [54], Chinese A-share-listed firms [55], and Malaysian firms [56]. The inclusion of SET ESG ratings as the independent variable reflects the growing importance of corporate sustainability in Thailand, offering a novel perspective within the context of an emerging economy. Additionally, several control variables—such as systematic risk (beta) [57], trading volume turnover [58], financial performance indicators, namely earnings per share, book value per share [59], profitability [60], leverage [61], firm size [62], cash holdings [63], audit quality [64], firm age [65] and dividend policy [66] (dividend yield and dividend payments)—are included based on their established influence on firm value in the fields of financial and corporate governance research. For instance, beta captures a firm’s market volatility, while profitability, firm size, and leverage are consistently linked to firm performance and are essential for isolating the specific effects of ESG ratings. A multiple regression model is employed to assess the impact of SET ESG ratings on firm value, controlling for other variables to mitigate potential confounding effects. This model is appropriate for the study as it enables a detailed examination of the unique contribution of SET ESG ratings while accounting for other influential factors. The regression assumes a linear relationship between the dependent and independent variables, as well as homoscedasticity in the error terms. Diagnostic tests, including tolerance and variance inflation factors (VIF), are applied to detect and address multicollinearity where necessary, thereby ensuring the robustness and reliability of the results.

**Table 1:** The variable definitions.

Symbols	Variables	Descriptions
**Panel A: Dependent variable**		
**FV**	Firm value	Natural logarithm of market capitalization.
**Panel B: Independent variable**		
**SETESG**	SET ESG ratings	Four SET ESG ratings, from "BBB" to "AAA," in order from the lowest to the highest ratings, were assigned numerical values from "1" to "4."
**Panel C: Moderator variables**		
**Single Third Party**	Single third-party ESG assessment	1 if the firm is assessed for ESG performance by a single third party, and 0 otherwise.
**Two Third Parties**	Two third-party ESG assessments	1 if the firm is assessed for ESG performance by two third parties, and 0 otherwise.
**Three Third Parties**	Three third-party ESG assessments	1 if the firm is assessed for ESG performance by three third parties, and 0 otherwise.
**Four Third Parties**	Four third-party ESG assessments	1 if the firm is assessed for ESG performance by four third parties, and 0 otherwise.
**Five Third Parties**	Five third-party ESG assessments	1 if the firm is assessed for ESG performance by five third parties, and 0 otherwise.
**ESG Book**	ESG assessment by ESG Book	1 if the firm is assessed for ESG performance by ESG Book, and 0 otherwise.
**Moody’s ESG Solutions**	ESG assessment by Moody’s ESG Solutions	1 if the firm is assessed for ESG performance by Moody’s ESG Solutions, and 0 otherwise.
**MSCI**	ESG assessment by MSCI	1 if the firm is assessed for ESG performance by MSCI, and 0 otherwise.
**LSEG**	ESG assessment by LSEG	1 if the firm is assessed for ESG performance by LSEG, and 0 otherwise.
**S&P Global**	ESG assessment by S&P Global	1 if the firm is assessed for ESG performance by S&P Global, and 0 otherwise.
**Panel D: Control variables**		
**BETA**	Systematic risk	The covariance between the returns of the stock and the market divided by the variance of the market returns.
**TVT**	Trading volume turnover	The total trading volume divided by total number of common shares outstanding and multiplied by 100.
**EPS**	Earnings per share	Net income divided by total number of common shares outstanding.
**BVPS**	Book value per share	Shareholder’s equity minus preferred equity divided by total number of common shares outstanding.
**ROA**	Profitability	Net income divided by total assets.
**LEV**	Leverage	Total liabilities divided by total assets.
**SIZE**	Firm size	Natural logarithm of total assets.
**CASH**	Cash holdings	Cash and cash equivalents divided by total assets.
**AQ**	Audit quality	1 if the firm is audited by a Big4, and 0 otherwise.
**DY**	Dividend yield	Dividend per share divided by price per share.
**AGE**	Firm age	Natural logarithm of firm age.
**DP**	Dividend payments	1 if the firm paid dividends in the year, and 0 otherwise.

To examine the impact of SET ESG ratings on firm value, this study estimates the following regression model, referred to as Model 1:

$${\mathrm{FV}}_{\mathrm{it}}=\beta_{0}+\beta_{1}{\mathrm{SETESG}}_{\mathrm{it}}+\beta_{i}{\mathrm{Control}}_{\mathrm{it}}+\mathrm{Industry}\;\mathrm{Dummies}+\varepsilon_{\mathrm{it}}$$ (1)

where, *FV*_*it*_ denotes the firm value of firm *i* in year *t*, utilizing the natural logarithm of market capitalization as a proxy for firm value. The variable *SETESG*_*it*_ represents the measure of corporate sustainability performance for firm *i* in year *t* as indicated by the SET ESG ratings. The control variables incorporated in the model are defined in detail in Table 1. Here, *β*_0_ signifies the intercept term, while *ε*_*it*_ represents the random error term. This model allows for a comprehensive analysis of the relationship between ESG performance and firm value while controlling for various firm characteristics. By utilizing this approach, this study aims to provide a more precise understanding of the influence of SET ESG ratings on firm value in the Thai market. It is expected that the SET ESG rating will have a positive effect on firm value, and, when combined with other control variables, the model will offer substantial explanatory power in explaining firm value.

In recent years, there has been an increasing focus on the measurement of ESG ratings by various international institutions, including ESG Book, Moody’s ESG Solutions, Morgan Stanley Capital International (MSCI), Dow Jones Sustainability Index (DJSI), Bloomberg ESG, LSEG (formerly Refinitiv), and S&P Global. While some Thai firms have already undergone ESG assessments by these third-party agencies, the implications for investors remain unclear. For example, a study investigating the effect of ESG performance on firm performance and market value across four ASEAN countries (Indonesia, Malaysia, Singapore, and Thailand) utilizing Bloomberg’s ESG Score found no significant impact on firm performance or market value, suggesting that ESG scores are not yet effectively integrated into performance evaluations [67]. Conversely, another study employing the same Bloomberg ESG Scores reported that improved ESG and financial performance enhanced the firm value of 19 commercial banks in the ASEAN region, including Thailand [68]. This discrepancy highlights that even ESG ratings from the same provider can yield varying results. Further research utilizing the MSCI ESG Score found a negative relationship with firm value, indicating that higher ESG scores may correspond to lower firm value in ASEAN nations, including Thailand [69]. Additionally, a study using Refinitiv ESG scores revealed significant adverse effects of ESG performance on firm value, profitability, and particularly on financing cash flows in Southeast Asian firms [70]. Another investigation focused on banks within ASEAN countries concluded that ESG ratings do not significantly impact market value [71]. The inconsistencies in ESG ratings from different agencies pose a considerable challenge, as greater disagreement among ratings can amplify forecast errors and dispersion among analysts, thereby exacerbating information asymmetry and leading to greater inaccuracies in forecasts [72]. Such misalignments undermine the robustness and reliability of ESG evaluation measures, potentially generating conflicting information that complicates investors’ assessments of portfolio sustainability. This situation may result in both the underestimation and overestimation of market opportunities for sustainable investments [73]. Furthermore, discrepancies in ESG ratings can impede socially responsible investing in firms [74] and have a pronounced negative effect on stock returns, diminishing investor confidence and leading to declines in stock performance [75]. Given these dynamics, ESG rating disagreement is closely linked to increased uncertainty within the capital market [76]. These discrepancies prompt critical inquiries into how third-party ESG assessments influence the relationship between SET ESG ratings and firm value. Further exploration of whether firms assessed by third-party ESG rating agencies experience different outcomes compared to those evaluated solely by SET ESG ratings could yield vital insights into the broader integration of ESG performance into firm value assessments in Thailand. To analyze the impact of SET ESG ratings on firm value and the moderating effect of third-party ESG assessments, this study proposes the following regression model, which will be referred to as Model 2:

$${\mathrm{FV}}_{\mathrm{it}}=\beta_{0}+\beta_{1}{\mathrm{SETESG}}_{\mathrm{it}}+\beta_{i}{\mathrm{SETESG}}_{\mathrm{it}}\;x\;\mathrm{Moderator}\;\mathrm{Variables}+\beta_{i}{\mathrm{Control}}_{\mathrm{it}}+\mathrm{Industry}\;\mathrm{Dummies}+\varepsilon_{\mathrm{it}}$$ (2)

The moderator variables in this study consist of ESG performance assessments from one to five distinct third-party evaluators, including ESG Book, Moody’s ESG Solutions, MSCI, LSEG, and S&P Global. This categorization enables an in-depth examination of how each third-party evaluation influences the relationship between SET ESG ratings and firm value. By incorporating these moderator variables, the research aims to provide valuable insights into whether investor responses to SET ESG ratings differ for companies that have also undergone external ESG assessments. It is hypothesized that, regardless of prior evaluations by third-party agencies, SET ESG ratings will maintain a positive influence on firm value. Additionally, this study investigates both the monthly and weekly effects of SET ESG ratings to evaluate the temporal strength of their impact on firm value. Through the analysis of these time intervals, the research aspires to offer a more nuanced understanding of how fluctuations in SET ESG ratings affect firm value over time, positing that the positive influence of these ratings will endure.

## Results

### Descriptive statistics

Table 2 presents the descriptive statistics for the variables, including the number of observations (N), minimum, maximum, mean, and standard deviation (SD).

**Table 2:** Descriptive statistics.

Variables	N	Minimum	Maximum	Mean	SD
**FV**	5809	7.098	13.836	10.066	1.541
**SETESG**	5809	1.000	4.000	2.720	0.916
**BETA**	5809	-0.110	2.500	0.955	0.500
**TVT**	5809	0.000	6.035	0.210	0.342
**EPS**	5809	-6.000	24.080	1.989	3.786
**BVPS**	5809	1.150	329.610	23.465	51.647
**ROA**	5809	-24.270	21.440	0.756	3.568
**LEV**	5809	0.066	0.891	0.526	0.190
**SIZE**	5809	21.337	29.118	24.380	1.819
**CASH**	5809	0.000	0.362	0.070	0.070
**AQ**	5809	0.000	1.000	0.830	0.442
**DY**	5809	0.000	46.440	3.744	4.737
**AGE**	5809	0.693	4.949	3.178	0.880
**DP**	5809	0.000	1.000	0.904	0.294

The analysis of the descriptive statistics reveals that the mean firm value is 10.066, with a standard deviation of 1.541. The mean SET ESG rating stands at 2.720, with a standard deviation of 0.916. The mean systematic risk is 0.955, with a standard deviation of 0.500. Trading volume turnover averages 0.210, with a standard deviation of 0.342. Earnings per share have a mean of 1.989, with a standard deviation of 3.786. Book value per share averages 23.465, with a standard deviation of 51.647. On average, the return on assets is 0.756, with a standard deviation of 3.568. The leverage has a mean of 0.526 and a standard deviation of 0.190. Firm size averages 24.380, with a standard deviation of 1.819. The cash holdings average 0.070, with a standard deviation of 0.070. Audit quality has a mean value of 0.830, with a standard deviation of 0.442. Dividend yield averages 3.744, with a standard deviation of 4.737. The average firm age is 3.178, with a standard deviation of 0.880, and the average dividend payment is 0.904, with a standard deviation of 0.294.

### Regression analysis

The regression analysis presented in Table 3 explores the determinants of firm value across multiple models, each incorporating distinct sets of variables. The results indicate that SET ESG ratings have a statistically significant positive impact on firm value (p < 0.001), with an adjusted R-squared of 19.60%. This suggests that firms with higher ESG performance are more likely to experience an increase in firm value [55]. Furthermore, all control variables demonstrate a significant effect on firm value (p < 0.001), implying that these factors exert a considerable influence on investor sentiment, which can lead to substantial fluctuations in firm value. Collectively, these variables contribute to explaining variations in firm values across the models, with the adjusted R-squared values reflecting the degree to which the predictors account for changes in firm values. Additionally, the results indicate the absence of multicollinearity among the independent variables, as evidenced by tolerance values exceeding zero and variance inflation factors (VIF) below 10. Consequently, this study incorporates all relevant variables to comprehensively test the research hypothesis.

**Table 3:** The predictive power of SET ESG ratings and firm-specific metrics on firm value.

Variables	FV	FV	FV	FV	FV	FV	FV	FV	FV	FV	FV	FV	FV	Tolerance	VIF
**SETESG**	0.443*** (37.610)													0.673	1.486
**BETA**		0.142*** (10.966)												0.733	1.364
**TVT**			0.191*** (14.792)											0.831	1.203
**EPS**				0.364*** (29.756)										0.573	1.746
**BVPS**					0.350*** (28.506)									0.544	1.837
**ROA**						0.100*** (7.671)								0.894	1.118
**LEV**							0.296*** (23.612)							0.586	1.706
**SIZE**								0.830*** (113.241)						0.797	1.254
**CASH**									-0.088*** (-6.769)					0.439	2.277
**AQ**										0.145*** (11.153)				0.895	1.118
**DY**											-0.133*** (-10.192)			0.843	1.187
**AGE**												0.062*** (4.735)		0.830	1.205
**DP**													0.183*** (14.200)	0.791	1.264
**Industry**	No	No	No	No	No	No	No	No	No	No	No	No	No	-	-
**Obs.**	5809	5809	5809	5809	5809	5809	5809	5809	5809	5809	5809	5809	5809	5809	5809
adj. R^2^	19.60%	2.00%	3.60%	13.20%	12.30%	1.00%	8.70%	68.80%	0.80%	2.10%	1.70%	0.40%	3.30%	-	-

**Note:** The t-statistics are calculated and reported in parentheses.

*** p < 0.001

** p < 0.01, and

* p < 0.05, respectively.

The regression analysis in Table 4 assesses the impact of SET ESG ratings on firm value, incorporating various control variables across different models. The findings consistently demonstrate a significant positive relationship between SET ESG ratings and firm value, with statistical significance at the 0.001 level in all models. The results indicate that firms with higher ESG performance, as measured by their ESG ratings, experience an increase in stock price [48]. Additionally, other significant predictors of firm value include systematic risk, trading volume turnover, earnings per share, return on assets, firm size, and audit quality, all of which are positively associated with firm value. Conversely, book value per share, leverage, cash holdings, dividend yield, and dividend payments exhibit negative effects on firm value. The adjusted R-squared values exhibit a progressive increase across the various regression models employed in this study, culminating in a significant level of 75%. This upward trend signifies that the incorporation of additional independent variables enhances the model’s explanatory power, thereby providing a more comprehensive understanding of the factors influencing firm value. The adjusted R-squared metric is particularly noteworthy as it adjusts for the number of predictors in the model, effectively penalizing the inclusion of extraneous variables that do not contribute meaningfully to the explanation of the dependent variable. A value of 75% indicates that a substantial portion of the variance in firm value can be accounted for by the independent variables included in the models. This finding not only affirms the robustness of the analytical framework but also underscores the relevance of the selected variables in capturing the complexities associated with firm value in the context of SET ESG ratings. Such a high adjusted R-squared value therefore enhances the overall credibility of the study. These findings underscore the importance of ESG performance and firm-specific metrics in determining firm value, highlighting the complex nature of value drivers in the context of ESG integration.

**Table 4:** SET ESG ratings and firm value.

Variables	FV	FV	FV	FV	FV	FV	FV	FV	FV	FV	FV	FV	FV
**SETESG**	0.443***	0.452***	0.447***	0.372***	0.351***	0.349***	0.332***	0.042***	0.048***	0.050***	0.067***	0.065***	0.069***
	(37.610)	(39.066)	(39.021)	(32.587)	(30.782)	(30.651)	(29.639)	(5.365)	(5.977)	(6.312)	(8.522)	(8.336)	(8.703)
**BETA**		0.169***	0.118***	0.164***	0.173***	0.173***	0.147***	0.070***	0.076***	0.077***	0.066***	0.070***	0.067***
		(14.563)	(9.667)	(13.793)	(14.754)	(14.792)	(12.648)	(9.372)	(9.968)	(10.202)	(8.839)	(9.303)	(8.826)
**TVT**			0.141***	0.110***	0.110***	0.112***	0.122***	0.067***	0.068***	0.066***	0.062***	0.058***	0.058***
			(11.560)	(9.353)	(9.436)	(9.642)	(10.698)	(9.087)	(9.225)	(9.059)	(8.642)	(8.093)	(8.027)
**EPS**				0.273***	0.183***	0.171***	0.149***	0.050***	0.052***	0.055***	0.052***	0.052***	0.053***
				(23.590)	(13.178)	(12.256)	(10.837)	(6.650)	(5.904)	(6.202)	(6.030)	(5.984)	(6.173)
**BVPS**					0.159***	0.169***	0.155***	-0.052***	-0.054***	-0.054***	-0.052***	-0.055***	-0.056***
					(11.497)	(12.218)	(11.353)	(-5.772)	(-5.947)	(-5.943)	(-5.877)	(-6.214)	(-6.364)
**ROA**						0.078***	0.070***	0.039***	0.035***	0.034***	0.023***	0.024***	0.029***
						(7.208)	(6.561)	(5.741)	(5.106)	(4.966)	(3.431)	(3.552)	(4.207)
**LEV**							0.172***	-0.196***	-0.206***	-0.206***	-0.223***	-0.229***	-0.233***
							(15.708)	(-24.102)	(-24.370)	(-24.402)	(-26.923)	(-26.997)	(-27.193)
**SIZE**								0.900***	0.897***	0.890***	0.890***	0.892***	0.897***
								(90.602)	(90.352)	(88.451)	(90.853)	(90.917)	(90.538)
**CASH**									-0.032***	-0.035***	-0.019**	-0.018*	-0.019**
									(-4.355)	(-4.644)	(-2.584)	(-2.519)	(-2.563)
**AQ**										0.027***	0.036***	0.038***	0.042***
										(3.923)	(5.330)	(5.586)	(6.125)
**DY**											-0.125***	-0.128***	-0.128***
											(-18.104)	(-18.389)	(-18.387)
**AGE**												0.023**	0.024***
												(3.148)	(3.389)
**DP**													-0.024***
													(-3.349)
**Industry**	Yes	Yes	Yes	Yes	Yes	Yes	Yes	Yes	Yes	Yes	Yes	Yes	Yes
**Obs.**	5809	5809	5809	5809	5809	5809	5809	5809	5809	5809	5809	5809	5809
adj. R^2^	19.60%	22.40%	24.10%	30.80%	32.30%	32.90%	35.60%	73.30%	73.40%	73.50%	74.90%	74.90%	75.00%

**Note:** The t-statistics are calculated and reported in parentheses.

*** p < 0.001

** p < 0.01, and

* p < 0.05, respectively.

The regression analysis in Table 5 explores the effect of SET ESG ratings on firm value, focusing on the moderating effect of third-party ESG performance assessments. The results consistently demonstrate that the interaction terms between SET ESG ratings and third-party evaluations yield varied outcomes. For example, firms assessed by a single third party exhibit a significant positive effect (p < 0.001), with an adjusted R-squared of 77.60%, suggesting that validation by a single third party amplifies the positive impact of SET ESG ratings on firm value. In contrast, firms evaluated by two third parties show a statistically significant negative effect (p < 0.001), with an adjusted R-squared of 75.10%, while those evaluated by three third parties display an insignificant negative effect (p > 0.5) with an adjusted R-squared of 75%. These results suggest that multiple third-party evaluations may diminish the positive influence of SET ESG ratings. Interestingly, the interaction terms for firms evaluated by four and five third parties return to significant positive effects (p < 0.001), with adjusted R-squared values of 75.50% and 76.60%, respectively, indicating a more complex dynamic between the number of third-party assessments and firm value. Further analysis of specific third-party evaluators reveals differing impacts. Interaction terms involving SET ESG ratings and evaluations by ESG Book, Moody’s ESG Solutions, MSCI, LSEG, and S&P Global all demonstrate significant positive effects (p < 0.001), with adjusted R-squared values of 77.10%, 75.60%, 77.50%, 76.90%, and 77.10%, respectively. These findings suggest that assessments from reputable third-party sources enhance the credibility and perceived value of a firm’s ESG performance, thereby positively influencing firm value. This aligns with prior research utilizing third-party ESG ratings, such as Bloomberg ESG Scores [68]. Overall, the analysis underscores the significance of both SET ESG ratings and external third-party validation in driving firm value, highlighting the role of third-party assessments in enhancing the reliability and impact of ESG performance.

**Table 5:** SET ESG ratings, firm value, and the moderating effect of third-party ESG performance assessments.

Variables	FV	FV	FV	FV	FV	FV	FV	FV	FV	FV
**SETESG**	0.065***	0.061***	0.065***	0.068***	0.061***	0.036***	0.057***	-0.011	-0.061***	-0.011
	(8.603)	(7.593)	(8.143)	(8.578)	(7.835)	(4.624)	(7.257)	(-1.345)	(-6.347)	(-1.272)
**SETESG x Single Third Party**	0.191***									
	(26.169)									
**SETESG x Two Third Parties**		-0.034***								
		(4.993)								
**SETESG x Three Third Parties**			-0.003							
			(0.478)							
**SETESG x Four Third Parties**				0.076***						
				(10.417)						
**SETESG x Five Third Parties**					0.153***					
					(19.696)					
**SETESG x ESG Book**						0.197***				
						(22.995)				
**SETESG x Moody’s ESG Solutions**							0.104***			
							(12.124)			
**SETESG x MSCI**								0.252***		
								(25.435)		
**SETESG x LSEG**									0.224***	
									(21.961)	
**SETESG x S&P Global**										0.273***
										(22.863)
**BETA**	0.054***	0.064***	0.066***	0.055***	0.072***	0.042***	0.070***	0.029***	0.062***	0.032***
	(7.440)	(8.336)	(8.537)	(7.119)	(9.654)	(5.709)	(9.286)	(3.785)	(8.447)	(4.274)
**TVT**	0.036***	0.059***	0.058***	0.051***	0.067***	0.056***	0.059***	0.044***	0.047***	0.036***
	(5.192)	(8.263)	(7.984)	(7.079)	(9.628)	(8.134)	(8.243)	(6.383)	(6.784)	(5.163)
**EPS**	0.052***	0.051***	0.052***	0.058***	0.016	0.024**	0.031***	0.050***	0.045***	0.056***
	(6.362)	(5.948)	(6.015)	(6.716)	(1.821)	(2.914)	(3.574)	(6.140)	(5.425)	(6.704)
**BVPS**	-0.006	-0.055***	-0.054***	-0.046***	-0.034***	-0.013	-0.047***	-0.010	-0.003	-0.006
	(-0.687)	(-6.152)	(-6.071)	(-5.219)	(-3.878)	(-1.512)	(-5.391)	(-1.179)	(-0.384)	(-0.662)
**ROA**	0.044***	0.027***	0.031***	0.030***	0.021***	0.035***	0.028***	0.029***	0.051***	0.026***
	(6.752)	(3.918)	(4.419)	(4.298)	(3.181)	(5.295)	(4.139)	(4.450)	(7.572)	(3.884)
**LEV**	-0.226***	-0.231***	-0.234***	-0.227***	-0.205***	-0.182***	-0.220***	-0.174***	-0.215***	-0.206***
	(-27.817)	(-26.968)	(-27.194)	(-26.701)	(-24.369)	(-21.380)	(-25.725)	(-20.558)	(-25.994)	(-24.836)
**SIZE**	0.811***	0.897***	0.898***	0.862***	0.821***	0.768***	0.839***	0.731***	0.810***	0.703***
	(81.661)	(90.852)	(90.138)	(83.127)	(79.488)	(69.601)	(76.997)	(64.008)	(78.637)	(55.129)
**CASH**	-0.033***	-0.020**	-0.021**	-0.021**	-0.025***	-0.011	-0.026***	-0.027***	-0.032***	-0.039***
	(-4.687)	(-2.669)	(-2.879)	(-2.849)	(-3.528)	(-1.632)	(-3.577)	(-3.838)	(-4.495)	(-5.526)
**AQ**	0.035***	0.039***	0.040***	0.040***	0.037***	0.036***	0.038***	0.030***	0.033***	0.037***
	(5.348)	(5.644)	(5.744)	(5.749)	(5.490)	(5.419)	(5.610)	(4.613)	(4.918)	(5.567)
**DY**	-0.112***	-0.128***	-0.124***	-0.134***	-0.110***	-0.150***	-0.115***	-0.135***	-0.115***	-0.146***
	(-16.555)	(-17.817)	(-17.300)	(-18.722)	(-15.794)	(-21.663)	(-16.260)	(-19.934)	(-16.740)	(-21.127)
**AGE**	-0.012	0.022**	0.024***	0.024***	0.007	0.005	0.006	0.007	0.000	-0.003
	(-1.766)	(2.998)	(3.360)	(3.375)	(1.006)	(0.759)	(0.758)	(1.036)	(-0.022)	(-0.428)
**DP**	-0.048***	-0.022**	-0.022**	-0.028***	-0.013	-0.020**	-0.015*	-0.034***	-0.041***	-0.040***
	(-6.840)	(-3.019)	(-2.966)	(-3.828)	(-1.813)	(-2.836)	(-2.069)	(-4.781)	(-5.673)	(-5.601)
**Industry**	Yes	Yes	Yes	Yes	Yes	Yes	Yes	Yes	Yes	Yes
**Obs.**	5809	5809	5809	5809	5809	5809	5809	5809	5809	5809
adj. R^2^	77.60%	75.10%	75.00%	75.50%	76.60%	77.10%	75.60%	77.50%	76.90%	77.10%

**Note:** The t-statistics are calculated and reported in parentheses.

*** p < 0.001

** p < 0.01, and

* p < 0.05, respectively.

The analysis in Table 6 investigates the monthly and weekly effects of SET ESG ratings on firm value, demonstrating a consistent and statistically significant positive impact across different time frames, which aligns with the signaling theory [36]. This pattern suggests that the positive influence of SET ESG ratings on firm value is sustained over time, emphasizing the importance investors place on ESG performance. Overall, the analysis confirms that SET ESG ratings announcements have a significant and enduring positive effect on firm value, with this effect remaining robust across various control variables and over different timeframes. During the month of the SET ESG ratings announcement, an adjusted R-squared of 75.10% indicates a substantial increase in firm value, and this effect remains significant in the first month following the announcement, with an adjusted R-squared of 74.50%. These findings suggest that the market responds favorably to the release of SET ESG ratings, resulting in an immediate and sustained boost in firm value (p < 0.001). On a weekly basis, the impact of SET ESG ratings is similarly pronounced. In the week of the announcement, the ratings have a positive and statistically significant effect on firm value (p < 0.01), with an adjusted R-squared of 75.70%. This positive effect persists in the following weeks, with adjusted R-squared values of 75.60% in the first week, 74.70% in the second week, 74.60% in the third week, 74.30% in the fourth week, 74.40% in the fifth week, 74.20% in the sixth week, and 74.10% in the seventh week.

**Table 6:** The monthly and weekly effects of SET ESG ratings on firm value.

Variables	The Month of SET ESG Ratings Announcement	1^st^ Month After the Month of SET ESG Ratings Announcement	The Week of SET ESG Ratings Announcement	1^st^ Week After the Week of SET ESG Ratings Announcement	2^nd^ Week After the Week of SET ESG Ratings Announcement	3^rd^ Week After the Week of SET ESG Ratings Announcement	4^th^ Week After the Week of SET ESG Ratings Announcement	5^th^ Week After the Week of SET ESG Ratings Announcement	6^th^ Week After the Week of SET ESG Ratings Announcement	7^th^ Week After the Week of SET ESG Ratings Announcement
**FV**	**FV**	**FV**	**FV**	**FV**	**FV**	**FV**	**FV**	**FV**	**FV**
**SETESG**	0.065***	0.066***	0.063**	0.064**	0.062**	0.066**	0.065**	0.067**	0.066**	0.069**
	(5.837)	(5.690)	(2.934)	(2.960)	(2.825)	(3.028)	(2.628)	(2.702)	(2.984)	(2.768)
**BETA**	0.058***	0.075***	0.043*	0.039	0.071***	0.073***	0.092***	0.050*	0.073***	0.061*
	(5.488)	(6.630)	(2.065)	(1.859)	(3.448)	(3.516)	(3.940)	(2.049)	(3.253)	(2.502)
**TVT**	0.077***	0.031**	0.112***	0.106***	0.057**	0.043*	-0.015	0.070**	0.035	0.066**
	(7.755)	(2.939)	(5.734)	(5.392)	(2.960)	(2.189)	(-0.685)	(2.924)	(1.643)	(2.887)
**EPS**	0.052***	0.054***	0.047*	0.047*	0.053*	0.056*	0.057*	0.044	0.056*	0.054*
	(4.306)	(4.322)	(2.040)	(2.012)	(2.242)	(2.356)	(2.128)	(1.645)	(2.347)	(1.997)
**BVPS**	-0.054***	-0.054***	-0.050*	-0.053*	-0.053*	-0.058*	-0.057*	-0.049	-0.054*	-0.056*
	(-4.364)	(-4.170)	(-2.076)	(-2.218)	(-2.189)	(-2.361)	(-2.073)	(-1.803)	(-2.195)	(-2.031)
**ROA**	0.030**	0.031**	0.032	0.029	0.031	0.033	0.032	0.030	0.031	0.027
	(3.131)	(3.054)	(1.705)	(1.570)	(1.634)	(1.730)	(1.507)	(1.396)	(1.619)	(1.278)
**LEV**	-0.229***	-0.236***	-0.227***	-0.225***	-0.237***	-0.235***	-0.243***	-0.230***	-0.235***	-0.235***
	(-19.042)	(-18.929)	(-9.874)	(-9.728)	(-10.135)	(-9.995)	(-9.138)	(-8.709)	(-9.812)	(-8.844)
**SIZE**	0.895***	0.897***	0.888***	0.896***	0.905***	0.896***	0.906***	0.891***	0.897***	0.894***
	(64.211)	(62.542)	(33.321)	(33.650)	(33.469)	(32.779)	(29.582)	(29.023)	(32.757)	(29.162)
**CASH**	-0.023*	-0.020	-0.021	-0.021	-0.022	-0.022	-0.017	-0.022	-0.019	-0.020
	(-2.271)	(-1.861)	***	(-1.037)	(-1.094)	(-1.080)	(-0.765)	(-0.957)	(-0.954)	(-0.861)
**AQ**	0.039***	0.041***	***	0.035	0.039*	0.041*	0.043*	0.037	0.040*	0.040
	(4.064)	(4.122)	***	(1.862)	(2.055)	(2.164)	(2.103)	(1.709)	(2.090)	(1.858)
**DY**	-0.122***	-0.125***	***	-0.121***	-0.121***	-0.124***	-0.127***	-0.121***	-0.125***	-0.126***
	(-12.242)	(-12.137)	***	(-6.287)	(-6.177)	(-6.306)	(-5.747)	(-5.457)	(-6.351)	(-5.686)
**AGE**	0.024*	0.026*	0.025	0.018	0.025	0.025	0.034	0.016	0.025	0.024
	(2.348)	(2.440)	(1.317)	(0.930)	(1.288)	(1.282)	(1.505)	(0.692)	(1.232)	(1.094)
**DP**	-0.020	-0.021	-0.021	-0.014	-0.025	-0.023	-0.021	-0.019	-0.023	-0.021
	(-1.909)	(-1.973)	(-1.072)	(-0.718)	(-1.232)	(-1.154)	(-0.941)	(-0.851)	(-1.130)	(-0.921)
**Industry**	Yes	Yes	Yes	Yes	Yes	Yes	Yes	Yes	Yes	Yes
**Obs.**	2983	2826	785	785	785	785	628	628	785	628
adj. R^2^	75.10%	74.50%	75.70%	75.60%	74.70%	74.60%	74.30%	74.40%	74.20%	74.10%

**Note:** The t-statistics are calculated and reported in parentheses. *** p < 0.001

** p < 0.01, and

* p < 0.05, respectively.

## Discussion and conclusion

This study investigates the influence of SET ESG ratings on firm value of companies listed on the Stock Exchange of Thailand, with a focus on three key dimensions: the overall effect of SET ESG ratings on firm value, the moderating role of third-party ESG performance evaluations, and the impact of SET ESG ratings following their initial announcement, thereby the summary of research results is shown in Table 7. The main findings indicate that firms with higher SET ESG ratings experience a notable enhancement in firm value, as these companies are perceived to be more proficient in managing environmental and social risks. This mitigates the likelihood of regulatory sanctions, litigation, and reputational damage. Consequently, such firms benefit from greater cash flow stability and a reduced cost of capital. Moreover, operational efficiencies stemming from ESG initiatives, including energy savings and resource optimization, lead to improved productivity and financial performance. A strong ESG reputation further enhances customer loyalty and attracts long-term investors, increasing demand for the firm’s stock. Firms with superior ESG ratings also benefit from improved access to capital, enjoying more favorable lending terms and opportunities for sustainability-linked financing, all of which contribute to higher firm value. These advantages help reduce uncertainty surrounding future stock risk and return [47], amplify investor reactions to positive ESG-related information [37], increase excess stock returns [1], and positively impact overall stock performance [46]. Additionally, previous studies confirm that ESG rating upgrades are associated with stock price increases [48], aligning with findings from other emerging markets such as Malaysia [56], Korea [54], and China [55].

**Table 7:** Summary of the research results.

Direct and moderating effects	Direction of effect	Statistical significance
**SET ESG ratings—- > Firm value**	Positive effect	Significant effect
**SET ESG ratings—- > Single third-party ESG assessment—- > Firm value**	Positive effect	Significant effect
**SET ESG ratings—- > Two third-party ESG assessments—- > Firm value**	Negative effect	Significant effect
**SET ESG ratings—- > Three third-party ESG assessments—- > Firm value**	Negative effect	Insignificant effect
**SET ESG ratings—- > Four third-party ESG assessments—- > Firm value**	Positive effect	Significant effect
**SET ESG ratings—- > Five third-party ESG assessments—- > Firm value**	Positive effect	Significant effect
**SET ESG ratings—- > ESG assessment by ESG Book—- > Firm value**	Positive effect	Significant effect
**SET ESG ratings—- > ESG assessment by Moody’s ESG Solutions—- > Firm value**	Positive effect	Significant effect
**SET ESG ratings—- > ESG assessment by MSCI—- > Firm value**	Positive effect	Significant effect
**SET ESG ratings—- > ESG assessment by LSEG—- > Firm value**	Positive effect	Significant effect
**SET ESG ratings—- > ESG assessment by S&P Global—- > Firm value**	Positive effect	Significant effect

The moderating effects of third-party ESG performance assessments provide valuable insights into how external validations interact with SET ESG ratings by illuminating the moderating role of third-party evaluations. The intricate interactions between SET ESG ratings and these evaluations reveal a multifaceted relationship that has not been thoroughly examined in the existing literature. By demonstrating that the impact of SET ESG ratings on firm value varies depending on the number of third-party evaluations. One of the most compelling insights from this research is the dual nature of third-party evaluations in influencing the relationship between SET ESG ratings and firm value. The analysis shows that validation from a single reputable third-party evaluator significantly enhances the positive impact of ESG ratings on firm value. This underscores the critical importance of credible endorsements in shaping investor perceptions. Firms can effectively leverage strong evaluations from trusted sources to enhance their market valuations, thereby highlighting the role of quality in third-party assessments. However, the findings also reveal a surprising twist: the negative impact associated with assessments from two third parties, alongside an insignificant negative effect when evaluated by three, suggests that more external validation can dilute the perceived value of ESG performance. This finding challenges the prevailing notion that multiple endorsements automatically strengthen a firm’s ESG profile, indicating instead that there is a threshold where the benefits of third-party validation may begin to wane. Such a realization prompts a reevaluation of how firms engage with third-party evaluators, stressing the need for a more strategic approach to seeking endorsements. The return to significant positive effects when firms receive evaluations from four or five third parties adds further complexity to the relationship between SET ESG ratings and firm value. This non-linear dynamic indicates that the influence of third-party evaluations may involve thresholds or diminishing returns, compelling firms to consider both the quantity and quality of the assessments they pursue. These findings highlight the necessity for firms to adopt a deliberate strategy in navigating third-party evaluations, ensuring that they understand the potential implications of their engagement with evaluators. Moreover, the study highlights the critical role of high-quality evaluations from renowned third-party sources such as ESG Book, Moody’s ESG Solutions, MSCI, LSEG, and S&P Global. Firms that successfully integrate strong ESG practices and secure credible external validation from these sources are more likely to experience enhanced market valuations, reflecting increased investor confidence in their sustainable business practices. The positive moderating effect of third-party evaluations on firm value is supported by prior research [68]. However, the negative moderating effect observed with evaluations from two to three third parties suggests that disagreement between ESG ratings can exacerbate information asymmetry, leading to greater inaccuracies and variability in forecasts [72]. This may result in the underestimation or overestimation of sustainable investment opportunities [73], as well as pronounced negative effects on stock returns [75]. These findings reinforce the notion that not all endorsements carry equal weight, and firms must carefully select which evaluations to prioritize in their ESG strategies. High-quality endorsements can significantly enhance a firm’s market standing, while excessive or conflicting assessments may undermine their value.

The findings also demonstrate that SET ESG ratings announcements lead to a significant and immediate increase in firm value, with this positive effect persisting beyond the announcement. This consistent impact highlights the crucial role of ESG performance metrics as key determinants of firm value, emphasizing the growing importance of sustainable practices in attracting investor interest. The notable market response following the announcement of SET ESG ratings indicates that investors are increasingly viewing ESG performance as a credible indicator of a company’s long-term viability and effective risk management. This aligns with signaling theory [36], which suggests that firms use credible signals to differentiate themselves in the market. In this context, SET ESG ratings serve as such signals, enabling firms to position themselves favorably in the eyes of investors. The study reinforces the relevance of ESG metrics and underscores their potential to enhance a firm’s reputation and investor trust, ultimately resulting in higher market valuations. Moreover, the sustained positive effects observed over several weeks after the announcement show that the initial market reaction is not transient but rather reflects a deeper, ongoing recognition of the value of robust ESG performance. This persistence suggests that companies with higher SET ESG ratings can maintain a competitive edge over time, fostering long-term investor confidence. It further supports the notion that consistent ESG performance contributes to long-term value creation. This insight adds a significant contribution to the existing literature by demonstrating that the impact of ESG ratings extends beyond immediate market reactions, encouraging firms to integrate ESG initiatives into their strategic planning. Another notable finding from the study is the implication that improving ESG performance is essential for firms aiming to enhance their market value. Incorporating ESG considerations into corporate decision-making signals a commitment to responsible, long-term investment practices [10], strengthens investor confidence, and improves the company’s reputation for financial transparency and reliability [13], ultimately increasing firm value [16]. In this regard, ESG ratings are no longer simply a score for firms and investors to monitor when deciding on additional capital financing. Instead, ESG metrics have evolved into tools that help both companies and investors identify growth opportunities, thereby driving long-term value creation [77]. In the Thai context, where sustainable development and responsible business practices are becoming more prominent, these findings provide essential guidance for firms to strategically engage with ESG principles, not only to meet regulatory requirements but also to secure a strong market position in an increasingly competitive and sustainability-focused business environment.

This study offers valuable insights into the impact of SET ESG ratings on the firm value of companies listed on the Stock Exchange of Thailand. However, it primarily captures short-term effects, leaving opportunities for future research to explore the long-term implications of SET ESG ratings. Future studies should examine how corporate sustainability performance, as reflected in SET ESG ratings, influences investor confidence over time. Additionally, researchers could investigate the non-linear dynamics identified in this study, particularly the threshold effects of third-party evaluations on firm value. Understanding when additional validation may become counterproductive could provide important insights into how companies manage external assessments. Furthermore, expanding the research to cover various sectors and geographic regions would offer a more comprehensive understanding of ESG performance and its impact across different markets. Addressing the limitations of this study can further enhance the reliability and generalizability of future findings. From a practical standpoint, the findings underscore the necessity for firms to prioritize the enhancement of their ESG performance and to strategically manage their relationships with third-party evaluators. Companies should recognize that high-quality endorsements from reputable third-party sources can significantly bolster their market valuations and enhance investor trust. Firms must adopt a targeted approach to engaging with external evaluators, ensuring that they pursue endorsements from sources known for their credibility and accuracy. Furthermore, companies should consider the potential pitfalls of reliance on third-party assessments, as more validation may dilute the perceived value of their ESG performance. This nuanced approach to managing ESG ratings can help firms navigate the complexities of investor perceptions and market dynamics effectively. The study also contributes to the theoretical landscape by reinforcing the importance of signaling theory in the context of ESG ratings. The findings illustrate how SET ESG ratings act as credible signals of a firm’s commitment to sustainable practices, thereby influencing investor perceptions and behaviors. This alignment with signaling theory suggests that firms can leverage ESG performance as a strategic tool for differentiation in the marketplace. Additionally, the research highlights the dual nature of third-party evaluations in shaping the relationship between ESG ratings and firm value, providing a foundation for future theoretical explorations into the dynamics of external validation. The non-linear relationship observed in the study offers a new perspective on how firms can strategically engage with third-party evaluators to maximize the benefits of ESG endorsements while minimizing potential drawbacks.

In summary, this research significantly contributes to the growing body of literature on ESG performance and firm value, particularly within the context of emerging markets such as Thailand. The findings affirm the critical role of ESG metrics in attracting investor interest and enhancing firm valuations. By integrating these insights into strategic planning and decision-making, firms can foster long-term value creation while navigating the evolving landscape of sustainable business practices. The insights gained from this study not only advance the understanding of the relationship between ESG performance and firm value but also provide a roadmap for companies aiming to enhance their market positioning in an increasingly sustainability-focused environment.

## Supporting information

S1 Table The variable definitions. (ZIP)

S2 Table Descriptive statistics. (ZIP)

S3 Table The predictive power of SET ESG ratings and firm-specific metrics on firm value. (ZIP)

S4 Table SET ESG ratings and firm value. (ZIP)

S5 Table SET ESG ratings, firm value, and the moderating effect of third-party ESG performance assessments. (ZIP)

S6 Table The monthly and weekly effects of SET ESG ratings on firm value. (ZIP)

S7 Table Summary of the research results. (ZIP)
